# Supplementary material for: Development of a Gene Delivery System of Oligonucleotides for Fibroses by Targeting Cell-Surface Vimentin-Expressing Cells with N-Acetylglucosamine-Bearing Polymer-Conjugated Polyethyleneimine
Source: Polymers (Basel). 2020 Jul 7;12(7):1508. doi: 10.3390/polym12071508 (PMC7407634; doi:10.3390/polym12071508)
Supplement: Supplementary file 1 [file polymers-12-01508-s001.pdf]

GPC chromatogram

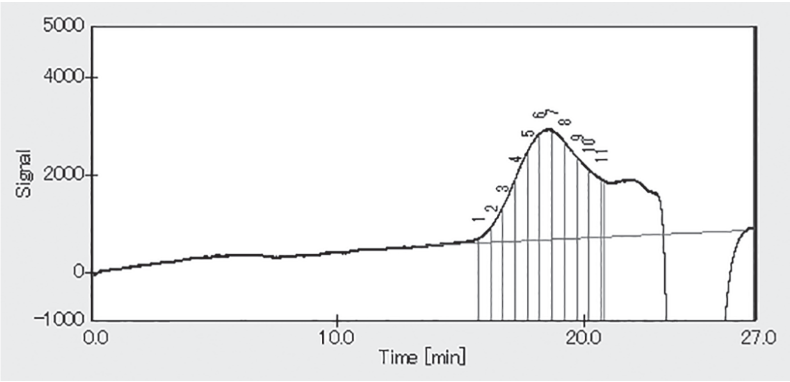

Number-average molecular weight (M_n) : 3400

Weight-average molecular weight (M_w) : 4700

M_w/M_n = 1.4

Calibration curve

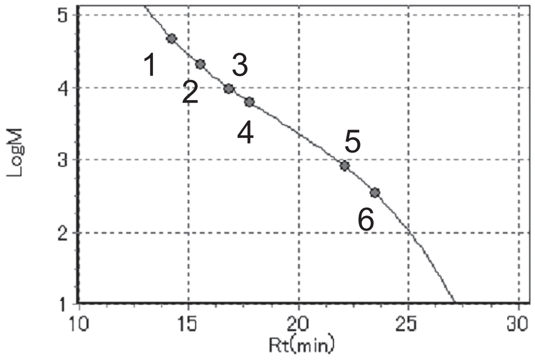

	Retention time (Rt) (min)	Molecular weight
1	14.233	47100
2	15.55	21100
3	16.883	9600
4	17.763	6100
5	22.117	828
6	23.477	342

Figure S1: GPC chromatogram of AC-GlcNAc. Calibration curve was produced based on a pullulan standard.

AC-GlcNAc-conjugated PEI (1 : 5)

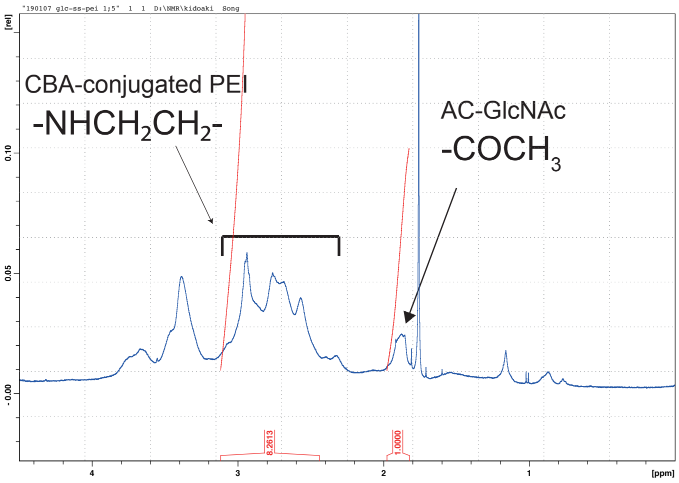

AC-GlcNAc-conjugated PEI (1 : 10)

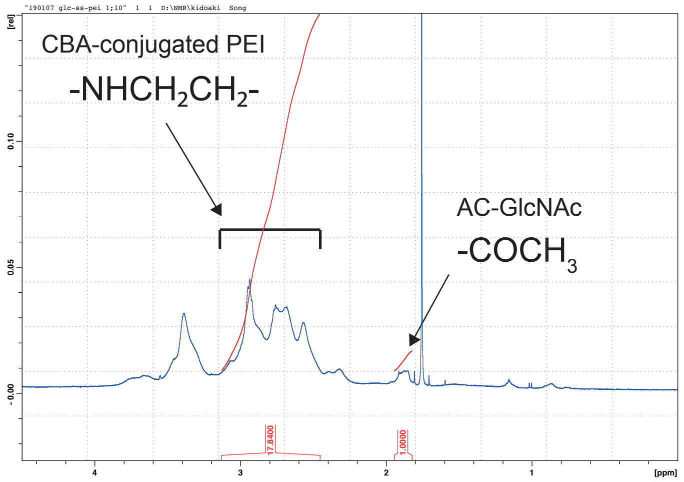

AC-GlcNAc-conjugated PEI (1 : 20)

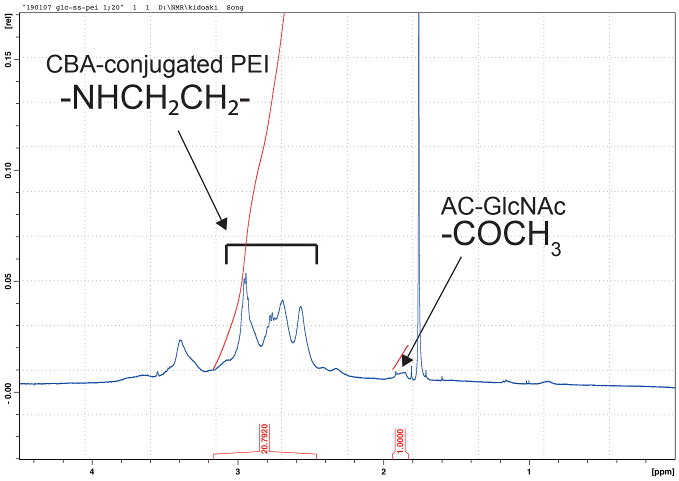

Figure S2: Nuclear magnetic resonance charts of AC-GlcNAc-conjugated polyethyleneimine (PEI) (1:5, 1:10, and 1:20).

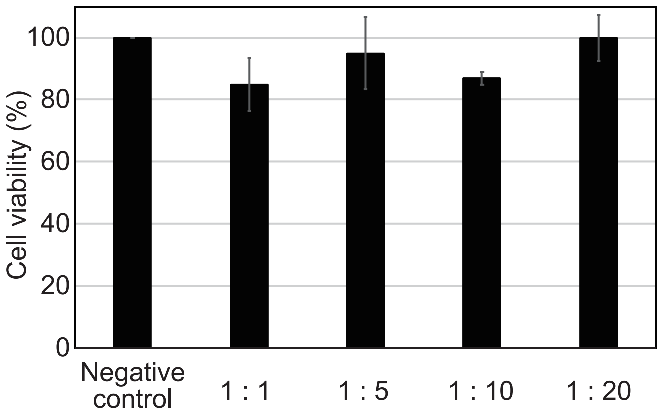

Figure S3: Evaluation of cell viability (%) of normal human dermal fibroblasts (NHDFs) after treatment with AC-GlcNAc-conjugated polyethyleneimine (PEI) complexes (1:1, 1:5, 1:10, and 1:20) based on a CCK-8 assay. n = 6
